# Risk factors for readmission among patients receiving outpatient parenteral antimicrobial therapy: a retrospective cohort study

**DOI:** 10.1007/s11096-022-01379-7

**Published:** 2022-02-14

**Authors:** Sabrine Douiyeb, Jara R. de la Court, Bram Tuinte, Ferdi Sombogaard, Rogier P. Schade, Marianne Kuijvenhoven, Tanca Minderhoud, Kim C. E. Sigaloff

**Affiliations:** 1 Department of Internal Medicine, Division of Infectious Diseases, Amsterdam UMC – Location VUmc, Amsterdam, The Netherlands; 2 Department of Medical Microbiology and Infection Prevention, Amsterdam University Medical Center, Amsterdam, The Netherlands; 3 Department of Clinical Pharmacology and Pharmacy, Amsterdam UMC – Location VUmc, Amsterdam, The Netherlands

**Keywords:** Antimicrobial stewardship, Antimicrobial therapy, Clinical pharmacology, Outpatient parenteral antimicrobial therapy

## Abstract

*Background* In the Netherlands, home treatment with intravenous antimicrobial therapy is a relatively new concept. Although several studies have shown that outpatient parenteral antimicrobial therapy (OPAT) can be administered safely, people receiving antimicrobials at home remain at risk for adverse events, including readmission. *Aim* The aim of our retrospective study was to identify risk factors for readmission in patients discharged with OPAT. *Method* Patients who were at least 18 years or older, discharged with OPAT between January 2016–December 2018 were included. Variables that were collected consisted of baseline demographics, complications, readmission within 30 days and treatment failure. Multivariate logistic regression analysis was performed to identify risk factors for readmission. *Results* A total of 247 patients were included; the most common reason for OPAT was bone and joint infections (17%). Penicillin (37%), cephalosporin (26%) and vancomycin/aminoglycoside (15%) were the most commonly prescribed antimicrobials. Among patients receiving medication subject to therapeutic drug monitoring (i.e. aminoglycosides or vancomycin), 51% (19/37) received weekly therapeutic drug monitoring. Receiving aminoglycosides or vancomycin (adjusted OR 2.05; 95% CI 1.30–3.25, *p* < 0.05) and infection of prosthetic material (adjusted OR 2.92, 95% CI 1.11–7.65, *p* < 0.05) were independent risk factors associated with readmission. *Conclusion* Although patients receiving medication subject to therapeutic drug monitoring are at higher risk of readmission, only half of the patients discharged with aminoglycosides or vancomycin were monitored according to IDSA guidelines. A specialized team in charge of monitoring patients with OPAT is more likely to increase the rate of monitoring to prevent readmissions and complications.

## Impacts on practice

- Discharge with vancomycin or aminoglycoside is an independent risk factor for readmission of OPAT-patients.
- In our setting, half of patients requiring therapeutic drug monitoring, received laboratory monitoring when discharged with OPAT.
- A specialized team in charge of monitoring OPAT-patients is more likely to increase the rate of therapeutic drug monitoring and could prevent readmissions and complications.

## Introduction

Ever since the introduction of outpatient parenteral antimicrobial therapy (OPAT), its application has increased [1]. OPAT can be administered when the patient is stable, but requires prolonged intravenous (IV) antibiotic therapy as a result of an active infection. In the past, OPAT has shown to be safe and to lead to a reduction of length of stay and subsequent decrease of costs [2, 3]. Previous studies have also described a high satisfaction rate of patients treated with OPAT at home [4, 5]. Although OPAT is usually considered safe, people receiving antimicrobials at home remain at risk for adverse events and readmission and therefore require appropriate follow-up and laboratory monitoring in the outpatient setting, when discharged with OPAT [6–9].

The Infectious Diseases Society of America (IDSA) 2018 guidelines recommend to have a specialized multidisciplinary team in place, an “OPAT-team”, consisting of an infectious disease specialist or pharmacist, in charge of coordinating the OPAT-care [10]. The introduction of a formal OPAT-team to coordinate the care of patients receiving intravenous antibiotic therapy at home can offer several benefits. First, a specialized team can assess the appropriateness of antimicrobials prescribed on hospital discharge and perform antimicrobial stewardship interventions if necessary [11, 12]. Second, the OPAT-team can monitor patients discharged with IV antibiotics and subsequently detect and potentially reduce adverse events and readmissions by intervening at an early stage [13–15].

Although consultation of the OPAT-team has been proposed for every patient before the initiation of OPAT, identifying patients with a high risk for readmission remains difficult. Previous studies have identified various risk factors to be associated with readmission, such as; discharge to a skilled nursing facility [7], previous hospital admission [9] and discharge with medication subject to TDM [6, 16].

### Aim

The aim of this study was to identify risk factors associated with readmission in patients receiving OPAT in a hospital without a formal OPAT-team. This information will be used for implementation and improvement of the OPAT-team program at a large teaching hospital in the Netherlands.

### Ethics approval

This study was approved by the Medical Ethical Committee (METC) of the Amsterdam UMC—location VUmc on the 15th of December 2020. Approval reference number: 2019.208.

## Method

### Patient population and setting

We performed a retrospective cohort study at the Amsterdam University Medical Center—location Vrije Universiteit Medical Center (VUmc), a 730-bed tertiary academic medical center in Amsterdam, the Netherlands. After approval of the study, a list of eligible patients discharged with OPAT in the period of January 2016 until December 2018 was generated by our hospital pharmacy. During this period a specialized OPAT team was not present in our hospital. Patients younger than 18 years of age were excluded, as were patients with missing data due to transfers to other hospitals or if hospital information was missing. Data were collected from the electronic patients records. Data was entered in a secured electronic database Castor.

### Data collection, definitions and outcomes

Variables were collected from documented notes from the electronic patient records (EPIC) and consisted of: patient demographics, comorbidities, date of admission and discharge, hospital ward, diagnosis, microbiological information, antimicrobial regimens and complications during outpatient treatment. OPAT days were calculated as the number of days between hospital discharge and the stop date of the antibiotic as noted in the patient records. Comorbidities were grouped in the following categories: diabetes mellitus, immune-compromised, malignancy, coronary heart disease and cognitive impairment. Information about the antibiotic regimen were collected and included type of received antimicrobial therapy and the start and stop date.

The primary outcome of this study was readmission within 30 days after discharge with OPAT. Risk factors for readmission were explored. Readmission was defined as readmission in the Amsterdam UMC—location VU within 30 days after discharge. Reasons for readmission and presentation at the emergency room were collected. Secondary outcomes included complications and monitoring frequency during OPAT. Complications were defined as antibiotic-related adverse drug events (ADE), line-related and non-OPAT related complications. Antibiotic-related adverse drug events consisted of decline in renal function, gastro-intestinal adverse events (loss of appetite, fatigue, malaise), hepatotoxicity, electrolyte imbalance and inadequate dosage of antimicrobial therapy (suboptimal or toxic doses). Line-related complications consisted of: mechanical issues (leakage, dislocation or dysfunction/obstruction of line of pump), line related infection and thrombosis.

The frequency of monitoring of the patient discharged with OPAT was recorded. Monitoring was defined as weekly contact defined as at least once a week, with the patient consisting of any of the following; telephone contact, laboratory testing or outpatient consultation. We analyzed the monitoring frequency in patients who received weekly monitoring versus who were not weekly monitored. In patients receiving medication subject to TDM (aminoglycosides and vancomycin), the frequency of therapeutic drug monitoring and the adherence to the IDSA guidelines were collected [10]. Therapeutic drug monitoring was considered to be adequate if a weekly blood sample was obtained. Treatment failure was defined as worsening or ongoing infection resulting in a visit to the emergency room or a readmission.

### Statistical analysis

Our cohort was obtained from a convenience sample of patients who received OPAT in the period of January 2016 until December 2018, in order to evaluate the quality and safety of care. No formal sample size calculation was performed. Categorical variables were summarized as proportions or frequencies, whereas continuous variables were summarized by median and interquartile range. A univariate and multivariate logistic regression were performed to identify risk factors associated with readmission. Variables with a *p *value < 0.20 in univariate analysis were included the multivariable logistic regression. A *p *value < 0.05 was considered statistically significant. Data were analyzed in IBM SPSS 26.

## Results

### Baseline characteristics and frequency of monitoring

**Fig. 1:**
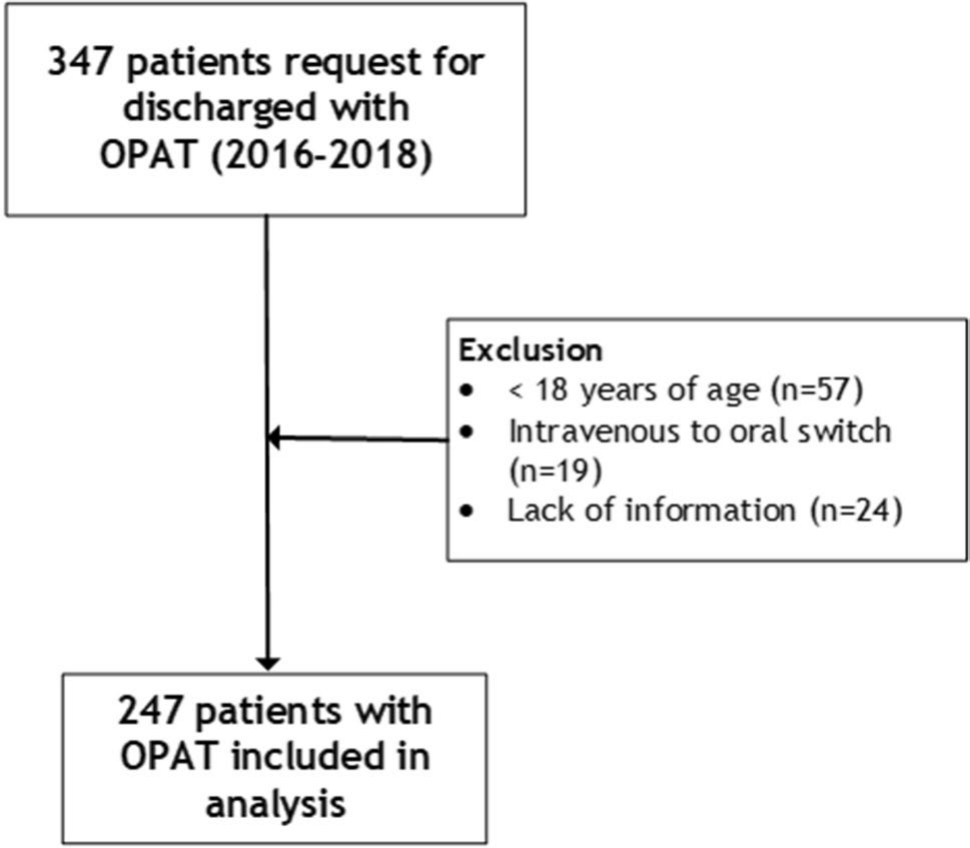
Inclusion of patients in the Amsterdam UMC-VU with OPAT treatment (2016–2018). Abbreviations: *OPAT* outpatient antimicrobial parenteral therapy

**Fig. 2:**
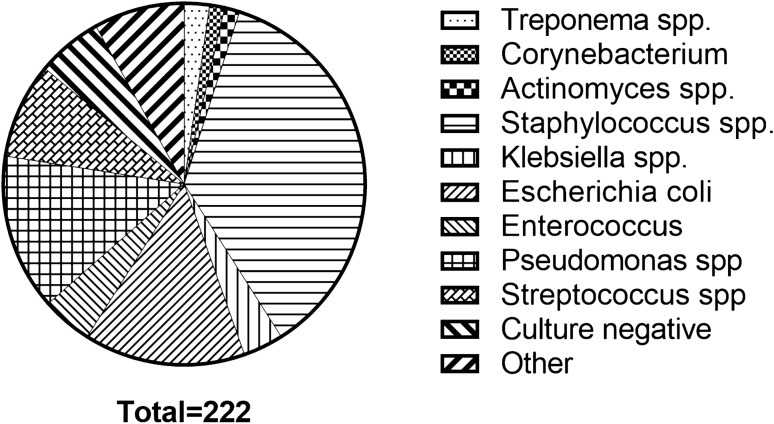
Distribution of bacterial pathogens in OPAT patients. Data are presented as No. (%) unless otherwise specified. Abbreviations: *spp* species

**Fig. 3:**
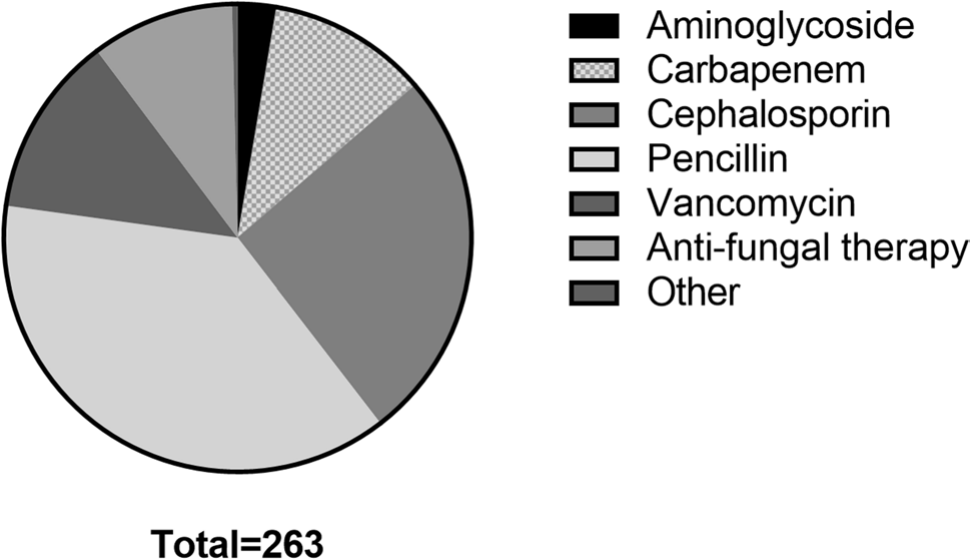
Distribution of parenteral antimicrobial therapy in OPAT patients. Data are presented as No. (%) unless otherwise specified

A total of 347 patients were discharged with between January 2016 and December 2018. Exclusion reasons are summarized in Fig. 1. A total of 247 patients were included for analysis, of which n = 27 were readmitted (Table 1). The majority of patients was discharged from non-surgical wards (56%). The median age was 62 years (IQR 50–72), 64% were male. Half of the patients (50%) had at least one comorbidity. The most prevalent indication for OPAT was osteomyelitis (17%), followed by prosthetic infections (14%), complicated urinary tract infections (14%) and respiratory infections (13%). The most frequent causative bacterial pathogen identified was *Staphylococcus aureus* (32%), followed by the *Pseudomonas spp*. (13%, Fig. 2). The majority of patients (84%) were discharged to receive OPAT through a PICC-line, the remaining were discharged with a peripheral infusion (11%) or central venous line (6%). The most common antibiotic group prescribed is penicillin (37%), followed by cephalosporins (26%) and vancomycin or aminoglycoside (15%, Fig 3).

**Table 1:** Baseline characteristics patients discharged with OPAT 2016–2018

	Total cohort (N = 247)	Not Readmitted (N = 220)	Readmitted N = 27	*p* value
*Hospital ward*				
Surgical	109 (44%)	95 (87%)	14 (13%)	0.417
Non-surgical	138 (56%)	125 (91%)	13 (9%)	
Age (median, IQR)	62 (50–72)	63 (50–72)	60 (50–72)	0.714
*Gender*				
Male	159 (64%)	145 (91%)	14 (9%)	0.151
*Indication for OPAT*				
Bone and joint infections	41 (17%)	36 (88%)	5 (12%)	0.785
Prosthetic infections(orthopedic)	35 (14%)	25 (71%)	10 (29%)	< 0.05
Complicated UTI	34 (14%)	32 (94%)	2 (6%)	0.551
Respiratory infections	32 (13%)	28 (88%)	3 (12%)	0.762
Vascular (stent) infections	22 (9%)	14	–	0.144
Intra-abdominal infection	18 (7%)	17 (94%)	1 (6%)	0.702
Central nervous system infection	17 (7%)	15 (88%)	2 (12%)	1.000
Skin and soft tissue infection	16 (7%)	14 (88%)	2 (12%)	0.689
Candidemia	15 (6%)	15	–	0.384
Endocarditis	11 (4%)	9 (82%)	2 (18%)	0.343
Bacteraemia (unknown focus of infection)	4 (2%)	4	–	1.000
Other	2 (1%)	2	–	1.000
*Antimicrobial therapy*				
Aminoglycosides/vancomycin	40 (15%)	28 (70%)	12 (30%)	0.000
Antifungal therapy	26 (10%)	26	–	0.053
Carbapenem	29 (11%)	28 (97%)	1 (3%)	0.331
Cephalosporin	68 (26%)	65 (96%)	3 (4%)	0.043
Penicillin	99 (37%)	87 (88%)	12 (12%)	0.679
Other	1(0.4%)	1		
OPAT treatment duration (median, IQR)	14 ( 8–32)	13 (7–32)	27 (11–36)	0.097
*Destination discharge*				
Nursing facility/rehabilitation center	28 (11%)	22 (79%)	6 (21%)	0.098
Home	219 (89%)	198 (90%)	21 (10%)	
*Type of line*				
PICC catheter	207 (84%)	183 (88%)	24 (12%)	0.586
Other catheter	40 (17%)	27 (68%)	13 (32%)	

*OPAT* outpatient antimicrobial parenteral therapy, *UTI* urinary tract infection

### Readmission

Twenty-five of the 247 patients were readmitted within 30 days (10%). The reasons for readmission and presentation at the emergency room are listed in Table 2. The majority of patients were readmitted due to treatment failure (44%) and line related complications (25%). Univariate analysis found that readmitted patients were more likely to have infection of prosthetic material and were more likely to be treated with vancomycin or aminoglycosides and were more likely to be treated for a longer duration. After adjusting for confounders in multivariate analysis (age, discharge destination, gender, treatment duration, indication) discharge with vancomycin or aminoglycoside (adjusted OR 2.05; 95% CI 1.30–3.25, *p* < 0.05) and infected prosthetic material (adjusted OR 2.92, 95% CI 1.11–7.65, *p* < 0.05) were found to be an independent predictor for readmission.

**Table 2:** Complications and readmission-rate during OPAT treatment

Complications	Total (n = 53)	Readmitted (n = 25)	Visit to emergency room (n = 4)
Adverse drug events	16	4 (16%)	1 (25%)
Catheter related events	21	6 (25%)	2 (50%)
Non-OPAT related	4	4 (16%)	–
Treatment failure	12	11 (44%)	1 (25%)

*OPAT* outpatient antimicrobial parenteral therapy

### Complications

A total of 41 patients (16%) experienced complications during OPAT treatment. Thirty-nine percent of complications consisted of antibiotic-related adverse drug events (ADEs) and 51% of line-related complications. ADEs consisted of gastro-intestinal adverse events (n = 6), electrolyte imbalance (n = 3), nephrotoxicity (n = 4), which were accompanied by toxic blood serum levels (n = 4), hepatotoxicity (n = 1) and subtherapeutic blood serum levels (n = 2).

Eight patients developed line related infections (38%). In two cases, patients developed a line thrombosis and eleven patients experienced dysfunction of the catheter. A total of twelve patients experienced treatment failure which consisted of worsening of ongoing infection resulting in a visit to the emergency room or re-admission (8% versus 92%).

### Frequency of monitoring

**Fig. 4:**
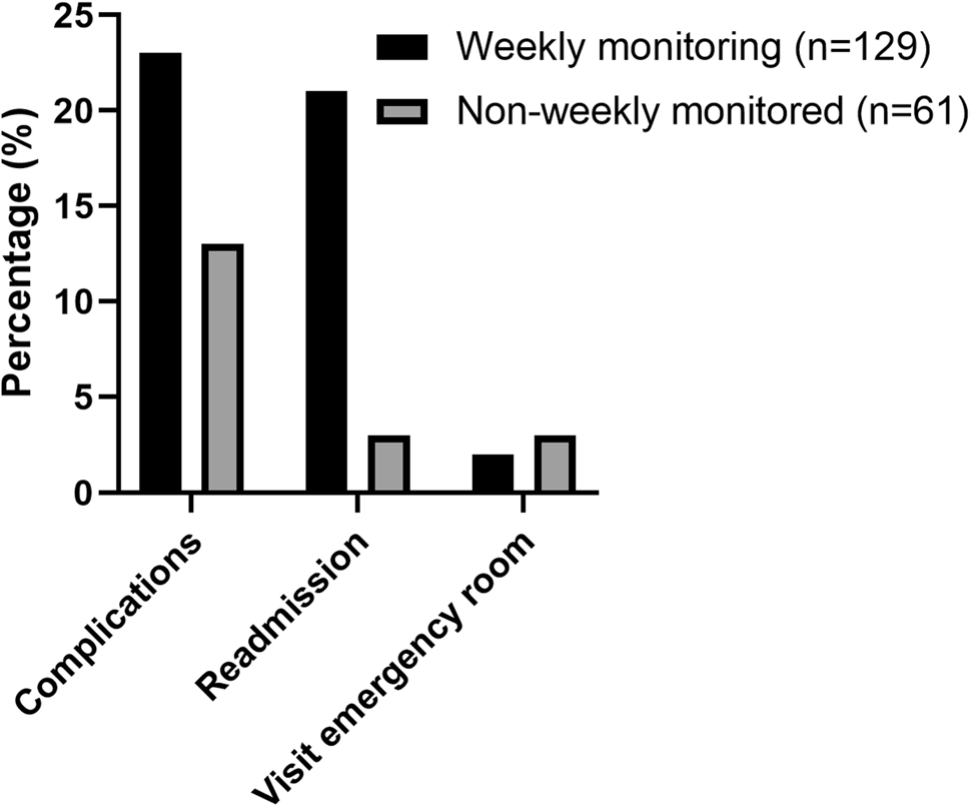
Complication and readmission rates based on frequency of monitoring. Data are presented as No. (%) unless otherwise specified

We assessed the frequency of monitoring in this study. A total of 57 patients were excluded from this analysis due to a OPAT duration of equal or less than 7 days, which did not require outpatient monitoring. Sixty-eight percent of the patients (129/190) were monitored on a weekly basis (see Fig. 4). We analyzed the rate of complications among those with weekly monitoring versus those who were not weekly monitored. Therapeutic drug monitoring was performed on a weekly basis in 51% (19 out of 37 patient receiving medication requiring TDM). Only 7 patients who received weekly therapeutic drug monitoring, met the complete laboratory recommendations of the IDSA guidelines for OPAT.

## Discussion

In this retrospective study we evaluated care for patients receiving OPAT, in a setting without a specialized OPAT-team in place, at a large teaching hospital in the Netherlands. Our main objective was to identify risk factors associated with readmission within 30 days after discharge. We found that patients discharged with vancomycin or aminoglycosides have a higher risk of readmission and only half of this group received TDM on a weekly base. Furthermore, infected prosthetic material was identified as a second risk factor for readmission. We found that a total 16% of the patients discharged with OPAT experienced complications.

Our first finding is in line with previous studies, which reported that patients treated with aminoglycosides [6] have a higher risk of readmission. Several studies have also found vancomycin to be a risk factor for readmission. [8, 16] One explanation for this finding is that aminoglycoside and vancomycin are nephrotoxic agents, which can cause kidney injury and therefore require close monitoring. Secondly, this study confirmed that infected prosthetic material to be a second independent predictor for readmission. This finding has previously been described by Duggal et al. [17], who observed that patients discharged with OPAT for prosthetic joint infections have a high readmission rate within 12 weeks of discharge (73%). This is the first study to identify infected prosthetic material as an independent predictor for readmission in OPAT-patients. A different study found discharge to a skilled nursing facility to be associated with a higher risk of readmission [7]. We were not able to confirm this finding in our study.

In this study we found that the majority of complications were line related (21/247). This is consistent with that of previous studies [11, 18]. Surprisingly, only 16 of the 247 (6%) patients were found to have antibiotic-related ADE documented. Previous studies evaluating the complication rate of antibiotic-related ADE in OPAT patients observed a higher complication rate of 15–20% [8], with one study even describing an ADE rate of 63% [19].

A possible explanation for this inconsistency may be the lack of adequate follow-up due to the retrospective nature of this study. We tried to correct for this factor by establishing the frequency of monitoring in this cohort. We observed that patients who were monitored on a weekly basis had a higher complication and readmission rate versus non-weekly monitored patients (23% versus 13% and 16% versus 5%). This could imply that complications go unnoticed in patients who did not receive weekly monitoring. This hypothesis is confirmed by Huck et al. [20], which found that less frequent monitoring, defined as non-availability of recommended test results, to be independently associated with readmission. In this study a broad definition of weekly monitoring was used, namely either telephone contact, laboratory testing or outpatient consultation). This discrepancy could lead to an overestimation of monitoring frequency.

In our study, therapeutic drug monitoring was performed poorly: only in 51% of cases, TDM was performed as indicated. Shah et al. [21] showed a similar therapeutic drug monitoring rate of 57% in the group of patients without ID supervision compared to a monitoring rate of 68.3% in patients with ID supervision. It is important that TDM is performed in these patients, due to the nephrotoxic effects of this group of antibiotics, when administrated in high doses. Intensive TDM offers the possibility to perform dosage adjustment and most likely prevent adverse drug events. In our center, a specialized OPAT-team responsible for the follow-up after discharge is not present.

One of the OPAT quality indicators is frequent laboratory monitoring, as recommended by the IDSA update 2018. In a recent Delphi study, Berrevoets et al. [22] tried to establish uniform advice for monitoring of laboratory results, which should be executed by the OPAT-team. Berrevoets et al. came to the conclusion that monitoring frequency of OPAT-patients should depend on the agent used, patients condition and comorbidities and duration of OPAT. This is in line with the recommendations of the IDSA guidelines. This study provides additional evidence in the risk factors for readmission in OPAT patients. Our findings emphasize the importance of appropriate monitoring in patients discharged with antimicrobials which require therapeutic drug monitoring. Additionally, introduction of an OPAT-team could lead to appropriate use of antibiotics, as previous studies have shown [11, 23], which could help increase antimicrobial stewardship.

This study contributes to understanding of the difficulties of OPAT. We identified independent risk factors in a large cohort of patients discharged with OPAT. These patients signify a respective proportion of the patients with OPAT. The results of our study highlight that OPAT teams should be aware of the higher risk of re-admission in this subgroup of patients. This study could be a great stepping stone for future research and improvement of OPAT-therapy.

Our study had several limitations. First, this study was a retrospective study. Due to the retrospective nature of this study, there remains a possibility that complications were underreported. Our study did give an overview of a large cohort of OPAT patients from 2016 until 2018. Subsequently, we also did not have access to any laboratory results outside of our academic hospital. This could underestimate the adherence to monitoring. An additional uncontrolled factor is the possibility of selection bias. Finally, this study was a single center observational study. This can lead to a lesser ability to generalizable our findings to other centers. However, OPAT is a relative new concept in the Netherlands, which are likely to experience the same obstacles.

In conclusion, this study contributes to understanding of the difficulties of OPAT. We identified independent risk factors in patients discharged with OPAT. These patients signify a respective proportion of the patients with OPAT. The results of our study highlight that OPAT teams should be aware of the higher risk of re-admission in this subgroup of patients. The results of this study underscore the need to implement IDSA guidelines and provide OPAT care on the basis of quality indicators, by means of a specialized multidisciplinary team for OPAT.

## Future research

Future research should focus on complications during OPAT and the frequency of monitoring especially TDM. Cost-effectiveness analysis should be integrated in future research in order to provide a solid business case for OPAT formal teams. Several questions, such as the appropriate quantity of laboratory monitoring, remain undetermined. More research using prospective trials is needed to answer these questions.
