# Correction to: Risk factors for readmission among patients receiving outpatient parenteral antimicrobial therapy: a retrospective cohort study

**DOI:** 10.1007/s11096-022-01393-9

**Published:** 2022-04-21

**Authors:** Sabrine Douiyeb, Jara R. de la Court, Bram Tuinte, Ferdi Sombogaard, Rogier P. Schade, Marianne Kuijvenhoven, Tanca Minderhoud, Kim C. E. Sigaloff

**Affiliations:** 1 Department of Internal Medicine, Division of Infectious Diseases, Amsterdam UMC – Location VUmc, Amsterdam, The Netherlands; 2 Department of Medical Microbiology and Infection Prevention, Amsterdam University Medical Center, Amsterdam, The Netherlands; 3 Department of Clinical Pharmacology and Pharmacy, Amsterdam UMC – Location VUmc, Amsterdam, The Netherlands

## Correction to: International Journal of Clinical Pharmacy https://doi.org/10.1007/s11096-022-01379-7

Unfortunately, the headings were not included in the initial version of this article's Abstract section. In this correction, the same was corrected.

The original article has been corrected.

